# Prechoroidal Cleft in Type 3 Neovascularization: Incidence, Timing, and Its Association with Visual Outcome

**DOI:** 10.1155/2018/2578349

**Published:** 2018-11-19

**Authors:** Jae Hui Kim, Young Suk Chang, Jong Woo Kim, Chul Gu Kim, Dong Won Lee

**Affiliations:** ^1^Department of Ophthalmology, Kim's Eye Hospital, Konyang University College of Medicine, Daejeon, Republic of Korea; ^2^Department of Ophthalmology, Konyang University College of Medicine, Daejeon, Republic of Korea

## Abstract

**Purpose:**

To investigate the incidence and timing of prechoroidal cleft development and its association with visual prognosis in type 3 neovascularization.

**Methods:**

This retrospective study included 166 eyes that were diagnosed with type 3 neovascularization. All eyes were treated with antivascular endothelial growth factor therapy. The incidence and timing of prechoroidal cleft development were evaluated. Best-corrected visual acuity (BCVA) at diagnosis and at final follow-up was compared between eyes with (cleft group) and without (no-cleft group) prechoroidal cleft. The incidence of retinal pigment epithelium (RPE) tear and subretinal hemorrhage was also compared between the two groups.

**Results:**

During the mean 39.7 ± 18.5 months of follow-up, prechoroidal cleft developed in 37 eyes (22.3%) at an average of 14.6 ± 10.4 months. The BCVA at final follow-up was significantly worse in the cleft group than in the no-cleft group (*P*=0.024), whereas the difference was not significant at diagnosis (*P*=0.969). The incidence of RPE tear (*P*=0.002) and subretinal hemorrhage (*P* < 0.001) was significantly higher in the cleft group.

**Conclusions:**

Prechoroidal cleft is a frequently observed finding during the treatment course of type 3 neovascularization. Eyes with prechoroidal cleft are at high risk of RPE tear or subretinal hemorrhage and subsequently associated with poor prognosis.

## 1. Introduction

Neovascular age-related macular degeneration (AMD) is one of the primary causes of visual loss in developed countries [1]. Although the advent of antivascular endothelial growth factor (VEGF) therapy has revolutionized treatment for this condition, the long-term visual outcome is still limited in some patients [2–4].

Prechoroidal cleft is an optical coherence tomography (OCT) finding that is characterized by a hyporeflective space between hyperreflective materials in pigment epithelial detachment (PED) and Bruch's membrane [5–8]. The incidence of prechoroidal cleft varies between 8.1% and 10% in neovascular AMD [5, 8]. This finding has been postulated to indicate residual activities of the subretinal hyperreflective materials [5]. More importantly, prechoroidal cleft was reported to be associated with a high incidence of retinal pigment epithelium (RPE) tear [5] and poor visual prognosis [8].

Type 3 neovascularization [9], also called as retinal angiomatous proliferation [10], is a peculiar form of neovascular AMD that is characterized by intraretinal neovascularization. Since the incidence of prechoroidal cleft is higher in type 3 neovascularization than other subtypes of neovascular AMD, [8] investigating the clinical significance of this condition is especially important in type 3 neovascularization. To the best of the authors' knowledge, however, no previous studies have focused on this subject.

The purpose of the present study was to investigate incidence and timing of prechoroidal cleft development during anti-VEGF therapy for type 3 neovascularization. The association of prechoroidal cleft with visual prognosis was also evaluated.

## 2. Materials and Methods

This retrospective, observational case study was conducted at a single center (Kim's Eye Hospital, Seoul, South Korea). The study was approved by the Institutional Review Board of Kim's Eye Hospital and was conducted in accordance with the tenets of the Declaration of Helsinki.

### 2.1. Patients

The study included patients who had been diagnosed with treatment-naïve unilateral type 3 neovascularization between January 2010 and July 2016. Additional inclusion criteria were as follows: (1) patients who were followed up for at least 12 months and (2) patients who were treated with anti-VEGF monotherapy. The exclusion criteria were as follows: (1) a history of treatment for choroidal neovascularization, (2) severe media opacity precluding clear fundus photographs, and (3) a concomitant retinal vascular disorder (e.g., macroaneurysm, proliferative diabetic retinopathy, and central retinal vascular occlusion).

### 2.2. Examinations

At baseline (diagnosis), all patients underwent a comprehensive ophthalmological examination, including measurement of best-corrected visual acuity (BCVA) and 90 D lens slit lamp biomicroscopy. Fundus photographs, fluorescein angiography images, and indocyanine green angiography (ICGA) images were obtained using a combined confocal scanning laser ophthalmoscope and spectral-domain OCT. The OCT images were taken using Spectralis HRA-OCT™ (Heidelberg Engineering, Heidelberg, Germany), RS 3000™ (Nidek Co., Ltd., Tokyo, Japan), or Spectral OCT™ (Ophthalmic Technologies Inc., Toronto, Canada).

Type 3 neovascularization was diagnosed based on a previously suggested method [11] and identified based on OCT findings. Patients with characteristic hyperreflective lesions in the outer retinal layer associated with intraretinal edema with or without subretinal fluid or subretinal pigment epithelial fluid were diagnosed with type 3 neovascularization. The presence of type 3 neovascularization was further confirmed by angiography results if focal hyperfluorescence with late leakage was observed at the site of neovascularization. The images were analyzed by two independent examiners (J.H.K. and Y.S.C.).

**Figure 1:**
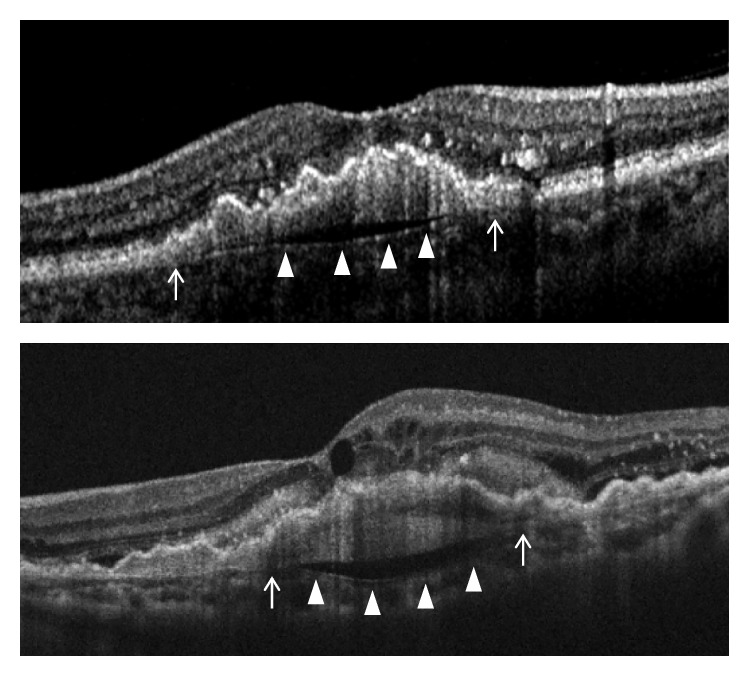
Representative images showing prechoroidal cleft (arrowheads), which developed during the treatment course of type 3 neovascularization. Note that the subretinal pigment epithelial tissue attaches to the Bruch's membrane at the edge of the cleft (arrows).

Staging of the lesion was performed based on the method suggested by Su et al. [12] as follows: stage 1 = intraretinal hyperreflective focus and cystoid macular edema (CME) without external limiting membrane (ELM)/ellipsoid zone (EZ) disruption; stage 2 = intraretinal hyperreflective focus, CME, and ELM/EZ disruption, with or without retinal pigment epithelium (RPE) disruption; and stage 3 = intraretinal hyperreflective focus, CME, ELM/EZ disruption, RPE disruption, and serous pigment epithelial detachment, with or without subretinal fluid. The prechoroidal cleft was identified based on OCT images as a hyporeflective space between hyperreflective materials in PED and Bruch's membrane (Figure 1).

### 2.3. Treatment and Follow-Up

The patients were initially administered 3 monthly injections of either ranibizumab (0.5 mg/0.05 mL of Lucentis™; Genentech, Inc., South San Francisco, CA, USA) or aflibercept (2.0 mg/0.05 mL of Eylea™; Regeneron, Tarrytown, NY, USA). After the initial treatment, the patients were scheduled to visit the hospital every 1–2 months. During the first 12 months, the follow-up interval was extended to 3 months at the physician's discretion. Subsequently, the follow-up interval was extended up to 4 to 5 months. Clinical examinations, OCT, and the measurement of BCVA were performed at each follow-up visit. Additional treatment was provided if any of the following changes were noted: (1) OCT evidence of persistent fluid 1–2 months after the previous injection; (2) reaccumulation of subretinal or intraretinal fluid involving or threatening to involve the fovea, as visualized using OCT; or (3) new or increased retinal or subretinal hemorrhage on clinical examination. Further treatment was administered using ranibizumab, aflibercept, or bevacizumab (1.25 mg/0.05 mL of Avastin^™^; Genentech). In some patients, the treatment regimen was changed from as-needed to a proactive regimen at the discretion of the treating physician.

The cost, benefits, and potential complications of the treatment were fully discussed with the patients. Further treatment was not performed when the patient refused despite sufficient explanation that their visual acuity could deteriorate and the chance to recover their vision may decrease without further treatment. Further treatment was also discontinued at the physician's discretion for cases in which it was not beneficial. Patients who discontinued treatment were then followed up every 2–5 months.

### 2.4. Outcome Measures

The incidence of prechoroidal cleft and the timing when prechoroidal cleft was first developed were evaluated. Patients were divided into 2 groups according to the presence of prechoroidal cleft: the cleft group and the no-cleft group. Characteristics, including age, sex, hypertension, diabetes mellitus, stage of disease, and follow-up period, were compared between the 2 groups. In addition, the BCVA at initial visit, at 12 months, and at final follow-up was compared between the 2 groups. When the patient failed to visit the hospital at exactly 12 months, data from the follow-up visit closest to that time point were used for analysis. The incidence of RPE tear and ≥1 disc area extent of subretinal hemorrhage was also compared. When RPE tear was accompanied with subretinal hemorrhage, the tear was considered to be the origin of hemorrhage and the eye was classified to exhibit RPE tear only. In the cleft group, the incidence of attachment between sub-RPE fibrovascular tissue and Bruch's membrane at the edge of the cleft (Figure 1) was evaluated.

The BCVAs were converted to the logarithm of the minimum angle of resolution (logMAR) for analysis. According to the suggestion by Holladay [13], the visual acuities of counting fingers and hand motion were converted to logMAR values of 2 and 3, respectively.

### 2.5. Statistics

The data are presented as the mean ± standard deviation where applicable. The statistical analyses were performed using a commercially available software package (SPSS version 12.0 for Windows; SPSS Inc., Chicago, IL, USA). Differences in the values between the 2 groups were analyzed using the chi-squared test, Fisher's exact test, or independent samples *t*-test with or without Bonferroni correction. *P* values <0.05 were considered to be statistically significant.

## 3. Results

**Figure 2:**
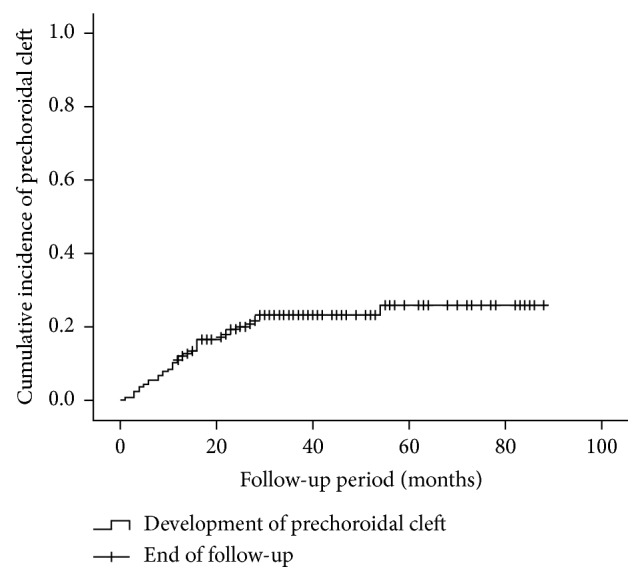
A Kaplan–Meier graph showing the cumulative incidence of prechoroidal cleft.

A total of 166 patients (166 eyes) met the eligibility criteria. The mean age was 75.4 ± 5.6 years, and the mean follow-up period was 39.7 ± 18.5 months. The prechoroidal cleft developed in 37 eyes (22.3%) at mean 14.6 ± 10.4 months after the diagnosis (Figure 2). In 31 of 37 eyes (83.8%), the cleft first developed within 24 months after the diagnosis, whereas the cleft developed after 24 months in the remaining 6 eyes (16.2%). In 30 eyes (81.1%), the prechoroidal cleft was accompanied with subretinal/intraretinal fluid. Attachment between sub-RPE fibrovascular tissue and Bruch's membrane at the edge of the cleft was identified in all 37 eyes. There was no significant difference in characteristics between the cleft group and the no-cleft group (Table 1).

In the cleft group (*n*=37), the mean number of anti-VEGF injection was 10.0 ± 3.6 throughout the follow-up period. Thirteen patients were treated with ranibizumab only and 4 patients were treated with aflibercept only. Four patients were treated with ranibizumab and aflibercept, and 14 patients were treated with ranibizumab and bevacizumab. The remaining 2 patients were treated using ranibizumab, aflibercept, and bevacizumab. The mean BCVA was 0.79 ± 0.27 (Snellen equivalent = 20/123) at diagnosis, 0.75 ± 0.43 (20/112) at 12 months, and 1.34 ± 0.61 (20/437) at final follow-up.

In the no-cleft group (*n* = 129), the mean number of anti-VEGF injection was 7.2 ± 3.6 throughout the follow-up period. Sixty-eight patients were treated with ranibizumab only, and 16 patients were treated with aflibercept only. Five patients were treated with ranibizumab and aflibercept, 35 patients were treated with ranibizumab and bevacizumab, and 4 patients were treated with aflibercept and bevacizumab. The remaining one patient was treated with ranibizumab, aflibercept, and bevacizumab. The values were 0.73 ± 0.34 (20/107), 0.64 ± 0.43 (20/87), and 1.02 ± 0.66 (20/209), respectively. BCVA was not significant different between the two groups at diagnosis (*P*=0.969) and at 12 months (*P*=0.711). However, the BCVA at final follow-up was significantly worse in the cleft group than in the no-cleft group (*P*=0.024). At final follow-up, the BCVA deteriorated ≥3 lines in 25 of 37 eyes (67.6%) in the cleft group, whereas the deterioration was noted in 61 of 129 eyes (47.3%) in the no-cleft group. Fourteen patients in the cleft group (37.8%) and 25 patients in the no-cleft group (19.4%) discontinued treatment during the follow-up period after full discussion with the patients. In the cleft group, treatment was not discontinued before the development of prechoroidal cleft in all eyes. The BCVA of all 39 patients who discontinued treatment was 20/400 or worse.

**Figure 3:**
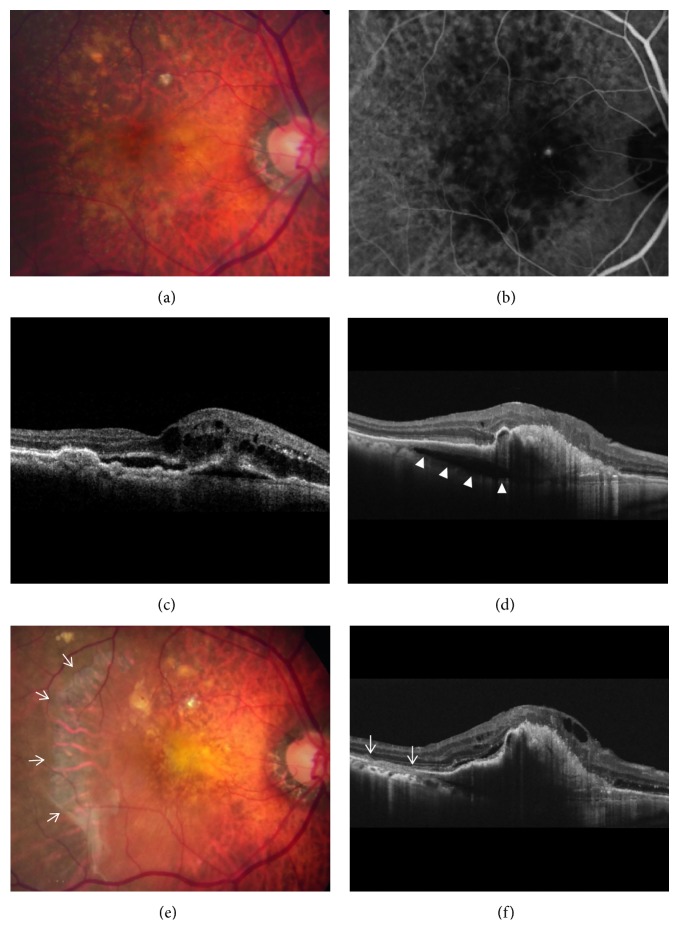
Fundus photography (a, e), indocyanine green angiography (b), and optical coherence tomography (c, d, f) images of a patient diagnosed with type 3 neovascularization. The images were taken at diagnosis (a–c), at 29 months (d), and at 35 months (e, f). The patient was treated with antivascular endothelial growth factor monotherapy during the follow-up period. Development of prechoroidal cleft was identified at 29 months (d) (arrowheads). Six months later, retinal pigment epithelium tear developed (e, f, arrows). Sixteen antivascular endothelial growth factor injections were performed during the follow-up period.

**Figure 4:**
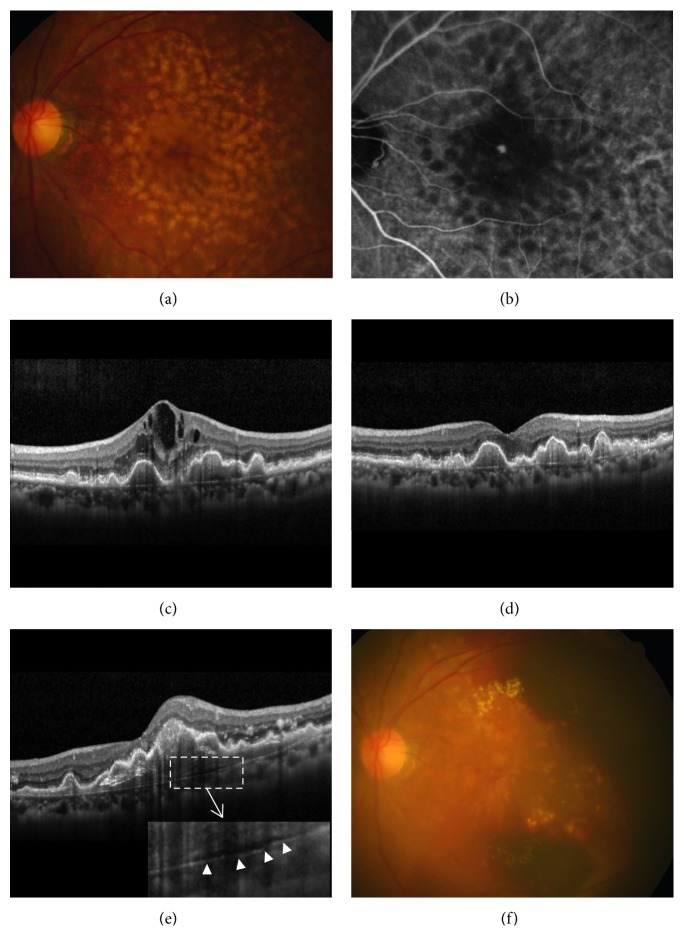
Fundus photography (a, f), indocyanine green angiography (b), and optical coherence tomography (c, d, e) images of a patient diagnosed with type 3 neovascularization. The images were taken at diagnosis (a–c), at 3 months (d), at 16 months (e), and at 17 months (f). After 3 monthly antivascular endothelial growth factor injections, the fluid was completely resolved (d). The patient was treated with anti-VEGF monotherapy during the follow-up period. Development of prechoroidal cleft was identified at 16 months (e) (arrowheads). One month later, subretinal hemorrhage developed (f). Nine antivascular endothelial growth factor injections were performed during the follow-up period.

In the cleft group, RPE tear (Figure 3) was noted in 8 eyes (21.6%) and subretinal hemorrhage (Figure 4) was noted in 10 eyes (27.0%). The mean period between the identification of prechoroidal cleft and the development of either RPE tear or subretinal hemorrhage was 6.0 ± 3.6 months. Among the 18 eyes with either RPE tear or subretinal hemorrhage, ≥3 lines of visual deterioration were noted in 15 eyes (83.3%). Among the remaining 19 eyes, ≥3 lines of visual deterioration were noted in 10 eyes (52.6%). In the no-cleft group, RPE tear was noted in 5 eyes (3.9%) and subretinal hemorrhage was noted in 6 eyes (4.7%). The incidence of RPE tear (*P*=0.002) and subretinal hemorrhage (*P* < 0.001) was significantly greater in the cleft group.

## 4. Discussion

In the present study, prechoroidal cleft developed in 22.3% of the patients with type 3 neovascularization at an average of 14.6 months after diagnosis. The majority of prechoroidal clefts first developed within 24 months after diagnosis. During the follow-up period, RPE tear or subretinal hemorrhage developed in approximately half of the eyes with prechoroidal cleft. As a result, the prognosis of eyes with prechoroidal cleft was poorer than that of eyes without the cleft.

In the study of Kim et al., prechoroidal cleft was associated with poor visual prognosis in neovascular AMD [8]. Poor response to initial therapy and uncontrolled submacular fibrosis were postulated as causes of this poor prognosis [8]. In the present study with type 3 neovascularization, we postulated that the high incidence of RPE tear and subretinal hemorrhage in the cleft group could be a primary cause of poor prognosis in the cleft group. RPE tear and subretinal hemorrhage are associated with poor prognosis in type 3 neovascularization [14–17]. Although the proportion of eyes with ≥3 lines of visual deterioration was higher in the cleft group (67.6%) than in the no-cleft group (47.3%), the proportion in eyes with prechoroidal cleft but without RPE tear or subretinal hemorrhage (52.6%) was similar to that of the no-cleft group.

**Figure 5:**
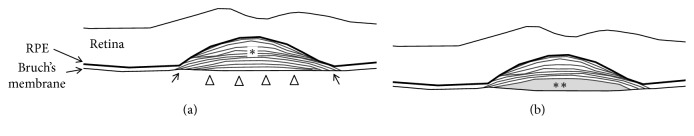
A schematic drawing showing the postulated developmental mechanism of prechoroidal cleft. (a) The subretinal pigment epithelial (RPE) fibrovascular tissue (asterisk) and Bruch's membrane may not attach or may be only weakly attached in some regions (arrowheads). However, around these regions, the two tissues attach firmly (arrows). (b) When sub-RPE tissue becomes contracted or leaks, prechoroidal cleft develops in the area of weak adhesion between tissues. The firmly attached area impedes further extension of the cleft.

The origin of prechoroidal cleft in neovascular AMD has been postulated to be contractile force of sub-RPE tissue and hydrostatic forces generated by leakage from the sub-RPE tissue [5, 6]. We additionally propose the following developmental mechanism of prechoroidal cleft in type 3 neovascularization (Figure 5). Unlike type 1 or type 2 neovascularization, type 3 neovascularization originally develops in the outer retina and then grows into the sub-RPE space [9, 11, 18]. Thus, it is possible that sub-RPE tissue and Bruch's membrane may not attach or only weakly attach in some regions. When sub-RPE tissue becomes contracted or leaks, prechoroidal cleft develops in the area of this weak adhesion between tissues. The firmly attached region impedes further extension of the cleft. In the present study, attached sub-RPE tissue to Bruch's membrane was noted in all 37 eyes with prechoroidal cleft, supporting this hypothesis.

**Figure 6:**
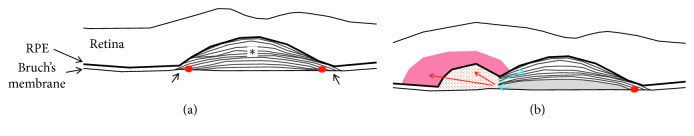
A schematic drawing showing the postulated developmental mechanism of subretinal hemorrhage in eyes with prechoroidal cleft. (a) The subretinal pigment epithelial (RPE) fibrovascular tissue (asterisk) firmly attaches to the Bruch's membrane in some regions (black arrows). In these regions, new vessels in the fibrovascular tissue may also firmly attach to the Bruch's membrane (red circles). (b) Strong sub-RPE tissue contraction or high-pressure exudation from the tissue (blue arrows) makes a force separating the sub-RPE tissue from the Bruch's membrane, resulting in rupture of vasculature, sub-RPE hemorrhage (area filled with red dots), and subretinal hemorrhage (area filled with light red).

We also postulated that the origin of hemorrhage might be closely associated with this firmly attached region. In this region, new vessels in the sub-RPE fibrovascular tissue may also firmly attach to Bruch's membrane. Contraction or leakage of sub-RPE tissue can make a force that separates the firmly attached new vessels from the Bruch's membrane, resulting in rupture of the vasculature (Figure 6). This mechanism may explain the higher incidence of subretinal hemorrhage in the cleft group than the no-cleft group.

Prechoroidal cleft is a frequently noted finding prior to RPE tear [19, 20]. In the study of Mukai et al., RPE tear developed in 23% of eyes with neovascular AMD exhibiting prechoroidal cleft [5]. In that study, cases of occult and classic CNV as well as retinal angiomatous proliferation were included, and most of the patients were treated with photodynamic therapy with ranibizumab [5]. In the present study, only type 3 neovascularization cases were included and all the patients were treated with anti-VEGF monotherapy. Nevertheless, the incidence of RPE tear was similar between the present study and the study of Mukai et al. In addition, eyes with prechoroidal cleft show a higher risk of RPE tear than eyes without it. This result suggests a close association between prechoroidal cleft and RPE tear. Nagiel et al. previously proposed a model for the development of RPE tear [19]; contraction of the CNV induces tensile forces that act in the plane of the RPE to cause a tear at the junction of the attached and detached RPE. If this hypothesis is valid, the close association of prechoroidal cleft with RPE tear is not surprising because contraction of sub-RPE fibrovascular tissue is one of the potential development mechanisms of prechoroidal cleft. It is possible that some contractile force of sub-RPE tissue exists in an eye that has presented with prechoroidal cleft at least once. RPE tear can develop owing to the aggravation of this force. In addition, we postulate that the characteristics of type 3 neovascularization may partially contribute to the high incidence of RPE tear in the cleft group. It is well known that age-related pathologic changes, including drusen, pseudodrusen, and choroidal thinning, are frequently observed in eyes with type 3 neovascularization [21–23]. Thus, it is possible that RPE cells are already compromised and less resistant to the tensile force inducing the tear.

Results of the present study suggest that patients exhibiting prechoroidal cleft should be closely monitored. Since RPE tear or submacular hemorrhage can cause abrupt and profound visual loss, it is desirable for clinicians to warn the patients regarding the risk of visual loss. In addition, considering the majority of the cleft develops within 24 months after the diagnosis, close OCT follow-up is needed during this period to identify the development of the cleft. Two important questions stemming from our observations are as follows: how to prevent the development of prechoroidal cleft and how to treat it effectively to avoid the development of catastrophic complications? In this retrospective study, unfortunately, a single strict retreatment guideline was not used. Thus, we could not evaluate the influence of the treatment strategy, such as fixed dosing, pro re nata, or treat-and-extend regimen, on the development or treatment outcome of prechoroidal cleft. Further studies with more controlled design are needed to address these questions.

The present study is to first focus on the clinical significance of prechoroidal cleft in type 3 neovascularization. However, this study has obvious limitations. This study was retrospective, and the analyses were performed based on real-world data. Since there was no strict follow-up and retreatment protocol, some of our patients may have been undertreated. Thus, the results of the present study may not be valid for patients who are treated with strict pro re nata or treat-and-extend regimens. In the present study, treatment was discontinued in 23.5% of the included patients. Treatment discontinuation is not rare among patients who undergo long-term treatment for neovascular AMD, and an unsatisfactory treatment outcome is one of the primary reasons [24, 25]. Although the reasons for treatment discontinuation were not accurately identified in this retrospective study, we postulate that the poor treatment outcome was a primary reason for relatively higher incidence of treatment discontinuation in the cleft group. In the present study, 77.7% of the patients were diagnosed with stage 3 disease using the OCT-based classification. This was relatively higher proportion than the previous study of Su et al. (67.6%) [12]. Thus, the incidence and timing of prechoroidal cleft found in the present study may not be valid for the cohort with earlier stage of disease. Lastly, since all patients were Korean, further studies are needed to verify whether our results are valid in other ethnic groups.

In summary, prechoroidal cleft developed in 22.3% of the patients who received anti-VEGF therapy for type 3 neovascularization. Eyes with prechoroidal cleft were at high risk of RPE tear or submacular hemorrhage and subsequently associated with poor visual prognosis. Further studies are required to establish better treatment strategies for this condition.

## Figures and Tables

**Table 1:** Comparison of characteristics between the cleft group and the no-cleft group.

Characteristics	Cleft group (*n*=37)	No-cleft group (*n* = 129)	*P* value
Age, years	74.7 ± 5.1	75.6 ± 5.7	0.377^*∗*^
Sex, no. (%)			0.457^†^
Men	7 (18.9)	32 (24.8)	
Women	30 (81.1)	97 (75.2)	
Hypertension, no. (%)	23 (62.2)	61 (47.3)	0.111^†^
Diabetes mellitus, no. (%)	10 (27.0)	28 (21.7)	0.497^†^
Phakia at diagnosis, no. (%)	27 (72.9)	72 (55.8)	0.061^†^
Stage of disease, no. (%)			0.146^†^
Stage 2	5 (13.5)	32 (24.8)	
Stage 3	32 (86.5)	97 (75.2)	
Cataract surgery during follow-up, no. (%)	2 (5.4)	9 (6.9)	1.000^‡^
Vitreoretinal surgery during follow-up, no. (%)	3 (8.1)	3 (2.3)	0.125^‡^
Follow-up period, months	39.1 ± 17.2	39.9 ± 18.9	0.836^*∗*^

Data are presented as the mean ± standard deviation where applicable. ^*∗*^Statistical analysis was performed using the independent samples *t*-test; ^†^statistical analysis was performed using the chi-squared test; ^‡^statistical analysis was performed using Fisher's exact test.

## Data Availability

The data used to support the findings of this study are restricted by the Kim's Eye Hospital IRB in order to protect patients' privacy. Data are available from Kim's Eye Hospital IRB for researchers who meet the criteria for access to confidential data.

## References

[1] Bourne R. R. A., Jonas J. B., Bron A. M. (2018). Prevalence and causes of vision loss in high-income countries and in Eastern and Central Europe in 2015: magnitude, temporal trends and projections. *British Journal of Ophthalmology*.

[2] Rofagha S., Bhisitkul R. B., Boyer D. S., Sadda S. R., Zhang K. (2013). Seven-year outcomes in ranibizumab-treated patients in ANCHOR, MARINA, and HORIZON: a multicenter cohort study (seven-up). *Ophthalmology*.

[3] Maguire M. G., Martin D. F., Ying G. S. (2016). Five-year outcomes with anti-vascular endothelial growth factor treatment of neovascular age-related macular degeneration: the comparison of age-related macular degeneration treatments trials. *Ophthalmology*.

[4] Pedrosa A. C., Sousa T., Pinheiro-Costa J. (2017). Treatment of neovascular age-related macular degeneration with anti-VEGF agents: predictive factors of long-term visual outcomes. *Journal of Ophthalmology*.

[5] Mukai R., Sato T., Kishi S. (2014). A hyporeflective space between hyperreflective materials in pigment epithelial detachment and Bruch’s membrane in neovascular age-related macular degeneration. *BMC Ophthalmology*.

[6] Rahimy E., Freund K. B., Larsen M. (2014). Multilayered pigment epithelial detachment in neovascular age-related macular degeneration. *Retina*.

[7] Mrejen S., Sarraf D., Mukkamala S. K., Freund K. B. (2013). Multimodal imaging of pigment epithelial detachment: a guide to evaluation. *Retina*.

[8] Kim J. M., Kang S. W., Son D. Y., Bae K. (2017). Risk factors and clinical significance of prechoroidal cleft in neovascular age-related macular degeneration. *Retina*.

[9] Freund K. B., Ho I. V., Barbazetto I. A. (2008). Type 3 neovascularization: the expanded spectrum of retinal angiomatous proliferation. *Retina*.

[10] Yannuzzi L. A., Negrao S., Iida T. (2001). Retinal angiomatous proliferation in age-related macular degeneration. *Retina*.

[11] Nagiel A., Sarraf D., Sadda S. R. (2015). Type 3 neovascularization: evolution, association with pigment epithelial detachment, and treatment response as revealed by spectral domain optical coherence tomography. *Retina*.

[12] Su D., Lin S., Phasukkijwatana N. (2016). An updated staging system of type 3 neovascularization using spectral domain optical coherence tomography. *Retina*.

[13] Holladay J. T. (2004). Visual acuity measurements. *Journal of Cataract and Refractive Surgery*.

[14] Kim J. H., Chang Y. S., Kim J. W. (2017). Difference in treatment outcomes according to optical coherence tomography-based stages in type 3 neovascularization (retinal angiomatous proliferation). *Retina*.

[15] Lee J. H., Lee M. Y., Lee W. K. (2017). Incidence and risk factors of massive subretinal hemorrhage in retinal angiomatous proliferation. *PLoS One*.

[16] Cho H. J., Kim H. S., Yoo S. G. (2015). Retinal pigment epithelial tear after intravitreal ranibizumab treatment for retinal angiomatous proliferation. *American Journal of Ophthalmology*.

[17] Kim J. H., Chang Y. S., Kim J. W., Kim C. G., Lee D. W. (2018). Early recurrent hemorrhage in submacular hemorrhage secondary to type 3 neovascularization or retinal angiomatous proliferation: incidence and influence on visual prognosis. *Seminars in Ophthalmology*.

[18] Li M., Dolz-Marco R., Messinger J. D. (2018). Clinicopathologic correlation of anti-vascular endothelial growth factor-treated type 3 neovascularization in age-related macular degeneration. *Ophthalmology*.

[19] Nagiel A., Freund K. B., Spaide R. F. (2013). Mechanism of retinal pigment epithelium tear formation following intravitreal anti-vascular endothelial growth factor therapy revealed by spectral-domain optical coherence tomography. *American Journal of Ophthalmology*.

[20] Mukai R., Sato T., Kishi S. (2014). Precursor stage of retinal pigment epithelial tear in age-related macular degeneration. *Acta Ophthalmologica*.

[21] Kim J. H., Chang Y. S., Kim J. W., Lee T. G., Kim C. G. (2015). Prevalence of subtypes of reticular pseudodrusen in newly diagnosed exudative age-related macular degeneration and polypoidal choroidal vasculopathy in Korean patients. *Retina*.

[22] Kim J. H., Kim J. R., Kang S. W., Kim S. J., Ha H. S. (2013). Thinner choroid and greater drusen extent in retinal angiomatous proliferation than in typical exudative age-related macular degeneration. *American Journal of Ophthalmology*.

[23] Cohen S. Y., Dubois L., Tadayoni R., Delahaye-Mazza C., Debibie C., Quentel G. (2007). Prevalence of reticular pseudodrusen in age-related macular degeneration with newly diagnosed choroidal neovascularisation. *British Journal of Ophthalmology*.

[24] Oishi A., Mandai M., Nishida A., Hata M., Matsuki T., Kurimoto Y. (2011). Remission and dropout rate of anti-VEGF therapy for age-related macular degeneration. *European Journal of Ophthalmology*.

[25] Vaze A., Fraser-Bell S., Gillies M. (2014). Reasons for discontinuation of intravitreal vascular endothelial growth factor inhibitors in neovascular age-related macular degeneration. *Retina*.

